# A Tale of Three Siblings: A Cluster of Toxic Neuropathy From a Less Suspected Source

**DOI:** 10.7759/cureus.115785

**Published:** 2026-09-04

**Authors:** Vivek Mishra, Jitupam Baishya, Hemanga Dhing, Masaraf Hussain, Riya Gautam

**Affiliations:** 1 Neurology, All India Institute of Medical Sciences Guwahati, Guwahati, IND

**Keywords:** arsenic toxicity, axonal polyneuropathy, bone marrow dysplasia, environmental exposure, megaloblastic anemia

## Abstract

Chronic arsenic exposure can affect multiple organ systems, notably the nervous system and hematopoietic tissue. We present three family members (two sisters and a brother) who developed subacute symmetric sensorimotor neuropathy with skin manifestations and anemia. Nerve conduction studies demonstrated axonal sensorimotor polyneuropathy. Urinary arsenic levels were markedly elevated in all three patients. The source of common exposure was found to be over-the-counter homeopathic medication that all three family members were taking. These cases underscore the importance of suspecting arsenic toxicity as a cause of unexplained neuropathy in appropriate settings.

## Introduction

Arsenic is a naturally occurring metalloid with significant toxic potential. Chronic exposure has been linked to multisystem effects including peripheral neuropathy, dermatologic changes, and bone marrow suppression [1-3]. Neurologically, chronic arsenic predominantly causes a symmetric, length-dependent axonal polyneuropathy affecting both sensory and motor fibers [4]. Hematologically, its interference with DNA synthesis and folate metabolism can result in macrocytosis or megaloblastic anemia [5,6]. Familial clustering of arsenic-induced toxicity is rare but may occur when household members share a common source. We report three family members (two sisters and a brother) who presented with subacute neuropathy, skin changes, and hematological abnormalities. All of them had elevated urinary arsenic levels.

## Case presentation

Case 1

The index case was a 48-year-old lady who presented with weakness of all four limbs for the last four weeks, which was insidious in onset and progressive in nature. It first started distally in both hands and feet, and over the period of 10 days, it involved the proximal upper and lower limbs. It was not associated with any difficulty in truncal weakness, breathing difficulty, or difficulty in eye movement. She also reported numbness, tingling, and burning sensations involving both upper and lower limbs for the same duration. Her complaints gradually progressed as she developed swelling of both lower limbs. There was no redness, warmth, pain, or features of deep vein thrombosis. On examination, she was conscious and oriented. Her vitals were within normal limits. She had hyperpigmentation over the gums (Figure 1A), both palms (Figure 1B), and the extensor surfaces of both arms and legs (Figure 1C), along with pallor. Mees’ line was absent. Her power in the upper limbs was 3/5 across all joints and 2/5 across all joints in the lower limbs. Sensory examination showed length-dependent impairment of all sensation up to the level of the knee joint and wrist joint. All deep tendon reflexes were in-elicitable. Plantar response was flexor. In view of the subacute nature of her peripheral neuropathy, common etiologies like immune-mediated, toxic, metabolic, and nutritional deficiency disorders were considered. Her routine blood examination showed macrocytic anemia with normal leukocyte and platelet counts. Her blood sugar, glycated hemoglobin, liver function, renal function, and thyroid profile were normal. Vitamin B12 was in the lower range (186 pg/mL), but viral markers and ANA were negative. Cerebrospinal fluid (CSF) analysis was unremarkable. Her nerve conduction study showed severe axonal sensorimotor neuropathy. Bone marrow examination showed dimorphic erythropoiesis, myeloid hypoplasia, and hypersegmented megakaryocytes. On toxicology analysis, urinary arsenic level was elevated (183 µg/day). 

**Figure 1:**
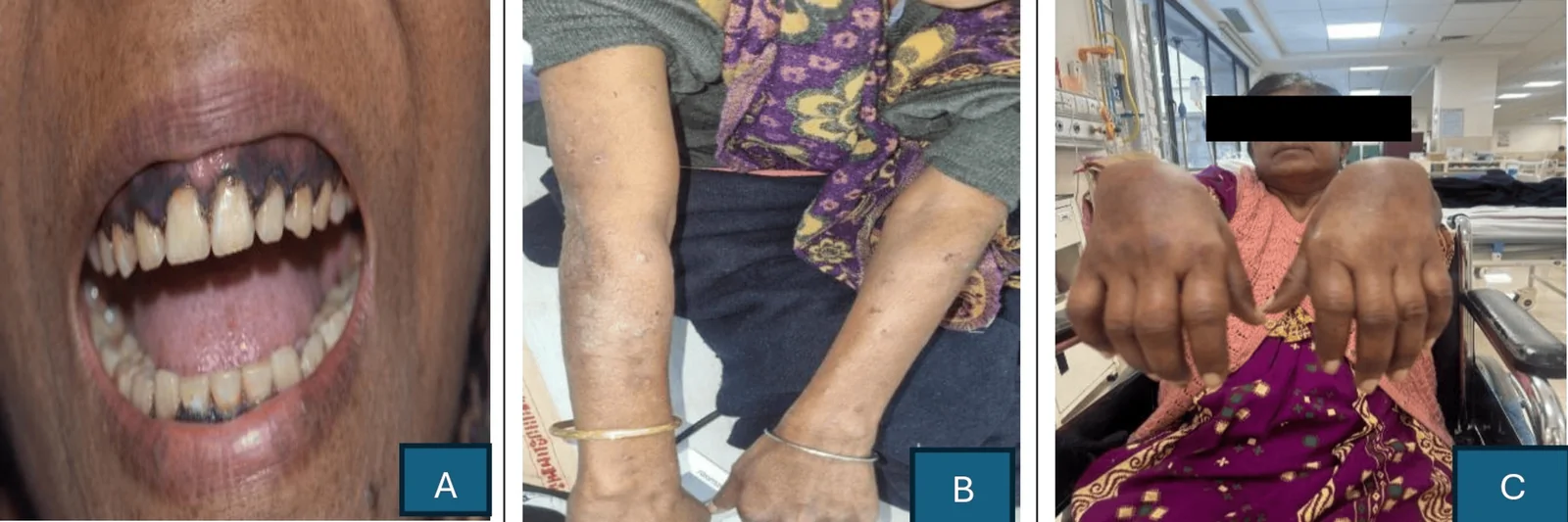
Hyperpigmentation over the gums (A), both palms (B), and the extensor surfaces of both arms and legs (C) in Case 1

Case 2

The second case was a 45-year-old lady, the younger sister of the index case, who developed similar symptoms concurrently with her elder sister, though with lesser severity. She also developed insidious-onset, gradually progressive weakness of all four limbs, which first started distally. Within 20 days, she could not lift her arms from the bed and had difficulty standing from a squatting position. It was associated with sensory loss up to the level of both ankle joints and wrist joints. On examination, she was conscious and oriented. She had bilateral pedal edema and hyperpigmentation over both palms and extensor surfaces of both upper and lower limbs (Figure 2A), as well as the gums (Figure 2B). Her power in the upper and lower limbs was 4/5 across all joints. Sensory examination showed graded impairment of all sensory modalities from distal to the proximal upper and lower limbs. She also had global areflexia and bilateral flexor plantar responses. Her routine blood examination showed macrocytic anemia with normal leukocyte and platelet counts. Blood sugar, glycated hemoglobin, liver function, renal function, and thyroid profile were normal. Vitamin B12 was in the low-normal range (282 pg/mL). Other etiological investigations like viral markers and ANA were negative. CSF analysis was not done because she was not willing. Her nerve conduction study showed severe axonal sensorimotor neuropathy. Toxicological analysis also showed an elevated urinary arsenic level (77 µg/day). 

**Figure 2:**
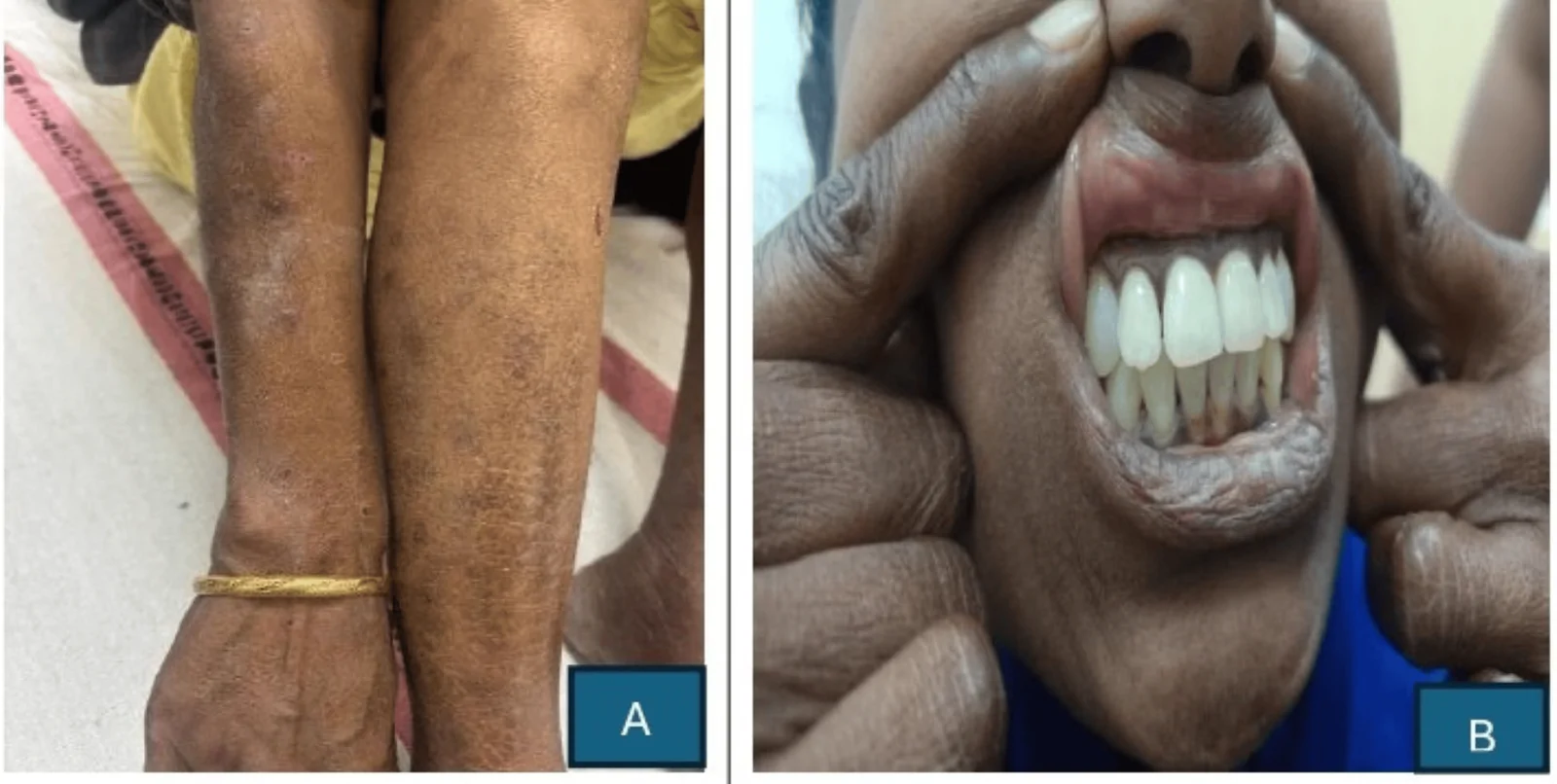
Hyperpigmentation over both palms and the extensor surfaces of both upper and lower limbs (A) and the gums (B) in Case 2

Case 3

Three months after the presentation of the first two cases, their 54-year-old brother was admitted to the intensive care unit with pneumonia and sepsis, for which he required mechanical ventilation for approximately 10 days. Following recovery from the acute illness and successful weaning from ventilatory support, he gradually developed weakness involving all four limbs over five days. The weakness was symmetrical in distribution and was more pronounced distally than proximally. In addition to motor weakness, the patient complained of sensory symptoms in the form of paresthesia and numbness in both the upper and lower limbs. The sensory symptoms were distributed in a glove-and-stocking pattern. On examination, he had hyperpigmentation over both legs, along with pallor. His power in the upper limbs was 3/5 across all joints, and in the lower limbs was 2/5 across all joints. Sensory examination showed length-dependent loss of all sensation. Deep tendon reflexes were absent in both the upper limbs and lower limbs. Plantar response was flexor. His routine blood examination showed mild anemia with normal leukocyte and platelet counts. Blood sugar, glycated hemoglobin, liver function, renal function, thyroid profile, and vitamin B12 were normal. Other etiological investigations like viral markers and ANA were negative. CSF analysis was not done because he was not willing. His nerve conduction study showed severe axonal sensorimotor neuropathy. Urinary arsenic levels were elevated (110 µg/day).

The laboratory and nerve conduction findings of all three cases are summarized in Table 1.

**Table 1:** Laboratory parameters and nerve conduction study of all three cases ALT - alanine aminotransferase; ANA - antinuclear antibody; AST - aspartate aminotransferase; B - basophils; CSF - cerebrospinal fluid; E - eosinophils; ESR - erythrocyte sedimentation rate; F - female; HbA1c - glycated hemoglobin; L - lymphocytes; M - male; M - monocytes; N - neutrophils; NCV - nerve conduction velocity; RBC - red blood cell; TSH - thyroid-stimulating hormone; WBC - white blood cell

Investigation	Normal values	Case 1	Case 2	Case 3
Hemoglobin	M: 13.5-17.5 g/dL; F: 12.0-15.0 g/dL	8.6 g/dL	7.9 g/dL	10.5 g/dL
Mean corpuscular volume	80-100 fL	112 fL	104 fL	96.6 fL
Mean corpuscular hemoglobin	27-33 pg	21.2 pg	26.4 pg	26.8 pg
Mean corpuscular hemoglobin concentration	32-36 g/dL	32.1 g/dL	33.2 g/dL	32.6 g/dL
Total leukocyte count	4000-11000/µL	5200/µL	7900/µL	6200/µL
Differential count (neutrophil/lymphocyte /monocyte/eosinophil/basophil) (%)	N: 40%-80%; L: 20%-40%; M: 2%-10%; E: 1%-6%; B: 0%-1%	72/21/3/3/1	82/10/4/3/1	65/28/3/2/2
Total platelet count	150-450 × 10^9^/L	208 × 10^9^/L	187 × 10^9^/L	262 × 10^9^/L
Total RBC	4.5-5.9 × 10^12^/L	4.38 × 10^12^/L	3.92 × 10^12^/L	5.18 × 10^12^/L
Peripheral smear	Normocytic, normochromic RBCs, no abnormal cells	Macrocytosis	Macrocytosis	Normocytic, normochromic
Serum vitamin B12	239-931 pg/mL	186 pg/mL	282 pg/mL	649 pg/mL
24-hour urinary arsenic level (µg/day)	<50 µg/day	183 µg/day	77 µg/day	110 µg/day
Bone marrow examination	-	Dimorphic erythropoiesis, myeloid hypoplasia, hypersegmented megakaryocytes	Not done	Not done
Serum creatinine	0.52-1.04 mg/dL	0.5 mg/dL	1.1 mg/dL	1.3 mg/dL
Random blood sugar	70-140 mg/dL	74 mg/dL	102 mg/dL	96 mg/dL
Serum AST	14-36 U/L	23 U/L	74 U/L	66 U/L
Serum ALT	<35 U/L	34 U/L	46 U/L	36 U/L
TSH	0.4-4.05 mIU/L	2.37 mIU/L	3.4 mIU/L	2.49 mIU/L
ANA	Negative	Negative	Negative	Negative
Viral serology	Negative	Negative	Negative	Negative
HbA1c	<6.5%	6.3%	5.4%	6.1%
Creatine phosphokinase	55-170 U/L	26 U/L	31 U/L	29 U/L
ESR	M: 0-20 mm/hour; F: 0-30 mm/hour	110 mm/hour	82 mm/hour	16 mm/hour
NCV findings	-	Axonal sensorimotor neuropathy	Axonal sensorimotor neuropathy	Axonal sensorimotor neuropathy
CSF cells	0-5 WBC/µL	2 WBC/µL	Not done	Not done
CSF sugar	40-70 mg/dL	77 mg/dL	Not done	Not done
CSF protein	12-60 mg/dL	55 mg/dL	Not done	Not done

Treatment

All three subjects received dimercaprol injections for two weeks as per the standard protocol [7]. Although there was no immediate symptomatic improvement, the progression of symptoms was halted, and nerve conduction parameters in Case 2 showed improvement of conduction parameters.

## Discussion

Arsenic toxicity is known to affect multiple organ systems, particularly the nervous and hematopoietic systems. Peripheral neuropathy associated with arsenic exposure is typically symmetric, length-dependent, and predominantly axonal, involving both sensory and motor fibers [4,8]. The patients in our series presented with progressive distal-to-proximal weakness and glove-and-stocking sensory deficits, findings that are consistent with the classical description of arsenic-induced neuropathy. The bilateral lower limb swelling observed in two of the cases may reflect arsenic-related vascular dysfunction or metabolic disturbances, which have been described in previous reports [6].

At the cellular level, trivalent arsenic (arsenite) exerts toxicity by binding to sulfhydryl groups in glutathione and key metabolic enzymes such as pyruvate dehydrogenase, thereby disrupting mitochondrial oxidative metabolism and promoting oxidative stress. These mechanisms ultimately lead to axonal degeneration and peripheral nerve damage [8]. Experimental animal studies have demonstrated structural alterations in peripheral nerves following arsenic exposure, supporting the clinical findings observed in affected patients.

Arsenic exposure may also result in significant hematological abnormalities, including macrocytosis, megaloblastic anemia, and dyserythropoiesis [5,6]. In the present series, bone marrow examination in Case 1 (index case) revealed dimorphic erythropoiesis, myeloid hypoplasia, and hypersegmented megakaryocytes, highlighting the toxic effects of arsenic on hematopoietic stem cells. Importantly, these hematological changes can occur even in the absence of vitamin B12 or folate deficiency, as arsenic interferes with DNA synthesis and folate-dependent methylation pathways [5].

Measurement of urinary arsenic levels serves as a sensitive biomarker for recent arsenic exposure, and all three siblings in this series demonstrated elevated urinary arsenic concentrations, confirming arsenic as the underlying etiology. The occurrence of similar clinical manifestations in the first two siblings suggested a shared environmental exposure. However, the diagnosis of arsenic neuropathy in the third sibling, who resides at a different geographical location and had no temporal relation to the presentation of the sisters, made common environmental source exposure less likely. Further probing revealed that all three subjects were self-prescribing and consuming the same homeopathic medications for a long duration. Certain traditional or alternative medicinal preparations have previously been reported to contain heavy metals, such as arsenic, either intentionally as part of traditional formulations or inadvertently through contamination. Therefore, illicit or unregulated use of homeopathic medication was suspected as the most plausible common source of arsenic exposure in these patients [9].

All three patients were administered the chelating drug dimercaprol. However, none of them showed any immediate clinical improvement, though progression of the symptoms has stopped. Chronic arsenic-induced neuropathy represents a predominantly axonal sensorimotor polyneuropathy in which structural nerve damage has already been established at the time of clinical presentation. In this context, chelation with dimercaprol (British anti-Lewisite (BAL)) has shown limited therapeutic efficacy. While BAL is effective in the acute phase of arsenic intoxication by binding circulating arsenic and facilitating its excretion, prior studies have consistently demonstrated that it does not produce clinically significant improvement in established neuropathy associated with chronic exposure [7]. This emphasized the need to diagnose these cases early for a better prognosis.

This case series highlights the importance of considering heavy metal toxicity in patients presenting with unexplained peripheral neuropathy accompanied by hematological abnormalities, particularly when there is clustering of cases within families. Early recognition and intervention - including cessation of the suspected exposure, chelation therapy where indicated, and supportive nutritional management - can lead to significant clinical improvement, although neurological recovery may be gradual and prolonged [10]. Furthermore, these cases emphasize the need for greater awareness regarding the potential risks of unregulated alternative medicinal preparations, which may contain toxic heavy metals and lead to significant systemic toxicity.

## Conclusions

The cases presented here highlighted the critical importance of recognizing arsenic’s dual toxicity on both the peripheral nervous system and the hematological profile. When multiple members of a single household present with similar, concurrent clinical features, it should immediately alert the clinician to a common source of exposure, such as alternative or homeopathic medications in the above cases. Patient outcome and prevention of permanent disability depend on urgent identification of the underlying etiology, removal of the source, administering targeted chelation, and providing aggressive supportive management.
